# Leeches

**Published:** 1857-04

**Authors:** 


### *Leeches.*

In the travels of Joseph Dalton Hooker, M. D., through Sikkim and Nepaul Himalayas, the following statements occur:—

"Leeches swarmed in incredible profusion in the streams and damp grass, and among the bushes: they got into my hair, hung on my eyelids, and crawled up my legs and down my back. I repeatedly took upwards of a hundred from my legs, where the small ones used to collect in clusters on the instep; the sores which they produced were not healed for five months afterwards, and I retain the scars to the present day. * * * * Another pest is a small midge or sand-fly, which causes intolerable itching and subsequent irritation, and is in this respect the most insufferable torment in Sikkim; the minutest rent in one's clothes is detected by the acute senses of this insatiable blood-sucker, which is itself so small as to be barely visible without a microscope. We daily arrived at our camping ground, streaming with blood, and mottled with the bites of peepsas, gnats, midges, and mosquitoes, besides being infested with ticks." (Vol. II, p. 18.) "A large tick infests the small bamboo, and a more hateful insect I never encountered. A traveler cannot avoid these insects coming on his person (sometimes in great numbers) as he brushes through the forest; they get inside his dress and insert the proboscis deeply without pain. Buried head and shoulders and retained by a barbed lancet, the tick is only to be extracted by force, which is very painful. I have devised many tortures, mechanical and chemical, to induce these disgusting intruders to withdraw the proboscis, but in vain."—(Vol. I, p. 166.)—*American Journal of Pharmacy.*

